# Dimensions of Pathological Aggression: From Neurobiology to Therapy

**DOI:** 10.3390/brainsci12030347

**Published:** 2022-03-03

**Authors:** Lisa Wagels, Ute Habel

**Affiliations:** 1 Department of Psychiatry, Psychotherapy and Psychosomatics, University Hospital Aachen, RWTH-Aachen University, 52074 Aachen, Germany; lwagels@ukaachen.de; 2 Institute for Neuroscience and Medicine (INM-10), JARA Institute Brain Structure-Function Relationship, Forschungszentrum Jülich, 52428 Jülich, Germany

## Introduction

This Special Issue brings together recent research on aggression on different scales, starting from animal models in low-aggression, healthy populations to patients with aggression problems. One of the most innovative tools to assess and reduce aggression is virtual reality, which was presented by two papers. Further intervention methods presented in this Special Issue were neurostimulation and behavioral therapy.

The first set of papers on neurobiological influence factors of aggression considered the neurobiological and behavioral correlates of aggression. Hormonal patterns associated with aggression were the topic of two papers. In the first paper, the simultaneous administration of arginine vasopressin and testosterone to healthy males resulted in increased aggressive behavior in unprovoked situations compared to the placebo group. Akkoc and colleagues pointed out that functional activity in the inferior frontal gyrus and inferior parietal lobule was enhanced during this competitive interaction in males whose hormonal levels were exogenously manipulated [1]. In a second paper on the hormone–aggression relationship, Pascual-Sagastizabal, del Puerto-Golzarri and Azurmendi showed that endogenous cortisol levels moderate authoritative parenting in fathers and aggression in their sons [2]. In contrast, permissive paternal parenting and girls’ aggressive behavior was moderated by negative emotionality. Muñoz Centifanti and colleagues highlighted the relevance of gaze shifting during fear recognition as a potential behavioral correlate of pathological aggression [3]. First, the authors demonstrated that fear recognition in children with high callous unemotional traits may be affected due to less frequent saccade shifting from the mouth to the eyes. Second, they successfully used specific eye gaze instructions to ameliorate deficits in recognizing fear recognition in children with callous unemotional traits. Further contributions to understanding pathological aggression was provided by studies concentrating on the neurobiological correlates of reactive and proactive aggression in overly aggressive groups. In their literature review, Belfry and Kolla presented key results on the biological correlates of proactive aggression, pointing to a blunted sympathetic functioning and, in addition to the amygdala, the involvement of the default mode network [4]. Such blunted physiological arousal was absent in an experimental study conducted by Harmsel and colleagues in a group of delinquent adults and controls [5]. Indeed, the authors reported a lack of group differences in psychophysiological and electrophysiological responses in reactive or proactive aggression. The final paper of this set of studies looked at a model for pathological aggression investigating the association of aggression with structural and functional degeneration in mice. Van Heukelum and colleagues showed that the midcingulate cortex was not activated during a resident–intruder test after massive structural degeneration [6]. Observing heightened levels of aggression in these mice, the authors highlighted the midcingulate cortex as a potential key area for pathological aggression.

The second set of studies referred to innovative techniques to assess and intervention methods to modulate aggression. In the first paper, Lobbestael and Cima presented newly developed virtual reality tasks for reactive and proactive aggression [7]. In the proactive aggression scenario, taking place in a virtual bar, participants could choose between physically fighting with an avatar or playing darts. Unfortunately, the scenario showed low validity, while the reactive aggression version, in which participants were first provoked by an avatar showed convergent validity with self-reported measures for aggression. The intervention methods presented in this Special Issue reveal a heterogeneous picture. Smeijers, Bulten, Verkes and Koole implemented a game for aggressive impulse management in virtual reality and tested this in a group of forensic outpatients [8]. The authors did not find the virtual game to be effective regarding aggressive behavior; yet, patients reported an increased insight into their own and others’ behavior, which already represents a valuable therapeutic outcome. Moreover, a mechanism-based anti-aggression psychotherapy, compared to a non-specific supportive psychotherapy, was able to reduce aggressive behavior. Neukel and colleagues elucidated in their study that the amygdala and the prefrontal cortex change their functional pattern [9]. Specifically, the pre-post treatment increase in connectivity between the amygdala and the dorsomedial prefrontal cortex in the mechanism-based anti-aggression psychotherapy compared to the non-specific psychotherapy group was highlighted as a potential brain mechanism underlying the response to threat cues. Finally, a literature review summarized the recent results concerning non-invasive brain stimulation methods as a tool to alter aggressive behavior. Knehans and colleagues concluded that against a large methodological heterogeneity, the significant effects in nine of eleven studies up- or downregulating aggression by using transcranial direct current stimulation or continuous theta burst stimulation on different prefrontal target regions are promising for interventions in clinical and forensic groups [10].
